# Biomimetic nanoparticles for targeted therapy of liver disease

**DOI:** 10.1039/d5pm00044k

**Published:** 2025-04-28

**Authors:** Veena Vijayan, Janitha M. Unagolla, Dhruvisha Panchal, Judith Eloyi John, Siddharth S. Menon, Jyothi U. Menon

**Affiliations:** a Department of Biomedical and Pharmaceutical Sciences, College of Pharmacy, University of Rhode Island Kingston RI 02881 USA jmenon@uri.edu; b International Academy East Troy MI 48083 USA; c Department of Chemical Engineering, University of Rhode Island Kingston RI 02881 USA

## Abstract

Liver fibrosis is a progressive and fatal condition characterized by stiffness and scarring of the liver due to excessive buildup of extracellular matrix (ECM) proteins. If left untreated, it can progress to liver cirrhosis and hepatocellular carcinoma (HCC)–one of the fastest-rising causes of cancer mortality in the United States. Despite the increased prevalence of liver fibrosis due to infections, exposure to toxins, and unhealthy lifestyles, there are no effective treatments available. Recent advances in nanomedicine can lead to more targeted and effective strategies for treating liver diseases than existing treatments. In particular, the use of biomimetic nanoparticles (NPs) such as liposomes and cell-membrane-coated NPs is of interest. NPs functionalized with cell membranes mimic the properties of the source cell used and provide inherent immune evasion ability, homologous adhesion, and prolonged circulation. This review explores the types of biomimetic coatings, different cargoes delivered through biomimetic NPs for various treatment modalities, and the type of core NPs used for targeting liver fibrosis and HCC.

## Abstract 2

Biomimetic nanoparticle formulations integrating different types of coatings, cores and cargoes for precise delivery of anti-fibrotic and anti-cancer therapies for the treatment of liver fibrosis and hepatocellular carcinoma.

## Graphical abstract

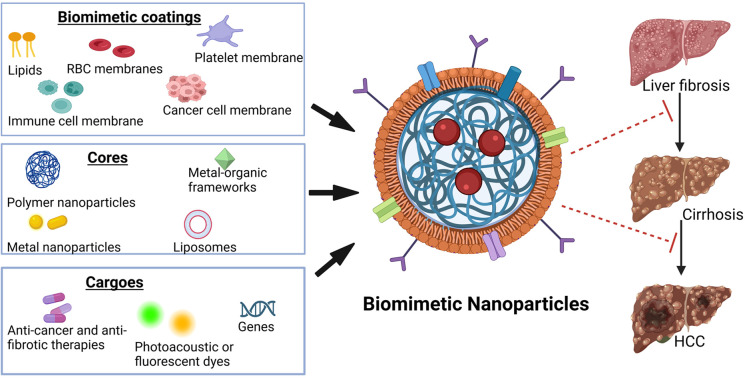

## 1. Introduction

As the primary organ involved in detoxification, protein synthesis and bile production, the liver is highly susceptible to the development of diseases especially when these processes are impaired or when there is damage or injury. An estimated 1.5 billion adults globally are currently affected by chronic liver disease.^1^ Per the American Liver Foundation, about 100 million people in the United States are currently living with some form of liver disease, and nearly 80–100 million adults are undiagnosed and unaware that they have fatty liver disease.^2^ Chronic liver conditions such as fatty liver disease, hepatitis, liver fibrosis, cirrhosis, and hepatocellular carcinoma (HCC) can have a detrimental effect on liver function leading to significant health problems. Liver fibrosis, characterized by the excessive buildup of extracellular matrix (ECM) proteins, usually manifests from chronic liver damage caused by hepatitis infections, alcohol misuse, or nonalcoholic fatty liver disease.^3^ This damage causes the liver to become stiff and scarred, impairing its ability to function effectively. Liver fibrosis tends to be an asymptomatic disease that progresses over time with no visible symptoms until advanced stages.^3^ Although there are currently no FDA-approved treatments against liver fibrosis, there are 100+ clinical trials assessing new treatments targeting the underlying causes and stages of liver fibrosis.^3^ If left untreated, liver fibrosis can progress to more dangerous conditions such as cirrhosis, and HCC; the latter affects approximately 800 million people worldwide with over 2 million deaths per year.^4^ HCC, the most common form of liver cancer, develops from hepatocytes, which are the parenchymal cells of the liver.^5^ Like liver fibrosis, HCC is also asymptomatic in the early stages, however, patients with more developed disease will face weight loss, fatigue, nausea, vomiting, satiety (a feeling of fullness despite eating very little food), and painful hepatomegaly.^6^ Treatments options for liver cancer include surgical resection, liver transplantation, and other localized treatments like radiofrequency ablation (heat generated by electrical current that is released in the tumor) and transarterial chemoembolization (a specific kind of chemoembolization used to block the short blood vessel that supplies oxygenated blood to the liver, also known as a hepatic artery, to treat the cancer).^6,7^ Systemic treatments such as immunotherapy and chemotherapy are also used. Despite the various treatment options available, liver cancer therapy continues to be a significant challenge, due to delayed identification, the aggressive nature of the disease, and the limited potency of current treatment options. These constraints emphasize the necessity for new, targeted strategies that address liver disorders more effectively.

Nanomedicine is a viable and promising approach to address the challenges associated with liver disease treatment. Nanoparticles (NPs), often sized at 10 to 1000 nm in diameter, can be used to transport therapies to specific cells, improving therapeutic efficacy, and minimizing side effects.^7^ Recent advancements in nanotechnology have made it possible to enhance the targeting capabilities of these formulations to the cells and sites of interest by using unique surface modifications, which helps reduce rapid clearance and potential off-target effects of the therapies. The reticuloendothelial system (RES), comprising phagocytic cells found primarily in the liver, spleen, lymph nodes and bone marrow, plays a crucial role in metabolizing and clearing NPs from the body. When NPs are designed to bypass the RES, they can avoid rapid clearance, remain in circulation longer, and as a result be able to accumulate in the specific cells and organs of interest for providing sustained therapy.

One of the methods by which NPs can avoid recognition by the immune system or clearance mechanisms is to coat them with materials (*e.g.* lipids, proteins, polysaccharides) that are intrinsic to the body. Such formulations, referred to as biomimetic NPs, are increasingly being explored for anti-cancer and anti-fibrotic drug delivery. Biomimetic NPs can be of three types: NP formulations surface modified with a cell-specific targeting ligand, NPs that are coated with cell membranes, and liposomes that are engineered with the cell membrane proteins of interest.^8,9^ Among these formulations, the cell membrane-coated biomimetic NPs (CM NPs) have received considerable attention in recent years as they can present a variety of complex biological proteins and molecules on the surface of the formulation impossible to achieve with traditional NP surface conjugations. Cells such as erythrocytes, macrophages and cancer cells have been explored widely for biomimetic NP-based drug delivery applications.^10^ As an example, Xia *et al.* recently developed biomimetic NPs coated with hepatic stellate cell (HSC) membranes presenting tumor necrosis factor-related apoptosis-inducing ligand (TRAIL) for liver fibrosis therapy. These NPs, with a diameter of about 227 nm, displayed selective targeting and efficient delivery of TRAIL to activated HSCs, which are key players in liver fibrosis progression, leading to their apoptosis.^11^ The cell membrane coating on the NPs enabled them to avoid the RES by providing a stealthy surface that reduces recognition by immune cells and prolongs circulation time in the bloodstream. Although a significant advantage of biomimetic NPs is their ability to express or retain properties of native cells to avoid rapid clearance, another key benefit is that they enable researchers to incorporate multiple therapeutics within the different layers of the formulation, for combinatorial treatment. In the above formulation, Xia *et al.* encapsulated all-trans retinoic acid (ATRA) within the polymer core of their biomimetic NPs, which worked synergistically with NP-delivered TRAIL to induce quiescence of those activated HSCs (aHSCs) that are TRAIL-resistant.^11^ In this review, we will discuss current research on the use of biomimetic NP formulations particularly in the context of liver fibrosis and HCC treatment, as these diseases pose significant global health challenges due to their rising incidence in recent years.

## 2. Pathophysiology of diseased liver and its challenges

Progression of chronic liver diseases, regardless of cause, is marked by a long history of chronic parenchymal injury, ongoing inflammation, and persistent liver fibrogenesis and wound healing responses.^12,13^ During liver fibrogenesis, the liver's functional tissue undergoes significant remodeling, characterized by the gradual accumulation of fibrillar ECM along with the nodular regeneration of liver tissue. If left untreated, liver fibrosis can progress to cirrhosis, leading to a gradual decline in normal liver function, which may ultimately result in liver failure and death.^14^ Advanced liver fibrosis and cirrhosis are also major risk factors for HCC. The main causes for liver injuries include chronic hepatitis B virus (HBV) and hepatitis C virus (HCV) infection, exposure to toxins (alcohol-related liver disease), metabolic dysfunction-associated steatohepatitis (MASH), and autoimmune diseases such as primary sclerosing cholangitis, primary biliary cirrhosis, and autoimmune hepatitis.

In liver fibrosis, the death of hepatocytes and cholangiocytes leads to the activation of HSCs. This activation can occur directly or through various cytokines released by immune cells, including innate lymphoid cells (ILCs), Kupffer cells, Th17 cells, and bone marrow-derived monocytes.^12^ One important cytokine is IL-17, which is secreted by Th17 cells. Elevated levels of IL-17 are found in conditions such as HBV and HCV, alcoholic liver disease, and autoimmune hepatitis.^15^ Additionally, neutrophils and mast cells can also significantly produce IL-17 in fibrotic liver tissue. IL-17 is known as a profibrogenic cytokine because it stimulates HSCs to increase the production of collagen type I, α-smooth muscle actin (α-SMA), and transforming growth factor-beta (TGF-β) by activating the nuclear factor kappa B (NF-κB) and signal transducer and activator of transcription 3 (STAT3) signaling pathways.^16^

The accumulation of the ECM due to liver injuries or wound healing process is sustained by the activation of hepatic myofibroblasts (MFs).^12,13,17^ The majority of the studies aiming at the origin of MFs have suggested that HSCs are most likely the main source of MFs in the injured liver.^18,19^ Other cell types, such as mesothelial cells and activated portal fibroblasts, also contribute to the pool of fibrogenetic MFs, though their contribution is minimal compared to HSCs, as suggested by several studies.^12,20,21^ In healthy livers, HSCs are found in the space of Disse, where they remain in a quiescent state and store vitamin A. However, after persistent liver injury, HSCs become activated and transform into myofibroblasts. During this process, they begin to express α-SMA, migrate to the site of tissue repair, and secrete large amounts of ECM. Interestingly, if the liver injury is resolved, myofibroblasts may undergo apoptosis and revert to an inactive state.^19^

Damaged and apoptotic hepatocytes prompt the activation of HSCs through two primary mechanisms: the release of damage-associated reactive oxygen species (ROS) and other fibrogenic mediators, as well as the recruitment of immune cells.^22,23^ These immune cells, in turn, facilitate HSC activation and promote collagen secretion by releasing cytokines and chemokines.^24,25^ After the initial activation of HSCs, they secrete cytokines that act in an autocrine manner, along with cytokines derived from immune cells. These signals help maintain the activation and survival of HSCs, as well as the deposition of ECM. Consequently, a vicious cycle develops, where mutual stimulation between inflammatory and pro-fibrogenic cells drives the process of hepatic fibrogenesis.^14,26,27^

Apart from the excessive deposition of ECM, significant changes in the quality and topographic distribution of ECM components occurs by altered remodeling and increased expression of tissue inhibitors of metalloproteinases (TIMPs).^13,17^ In a healthy liver, the ECM in the space of Disse—the area between endothelial cells and hepatocytes—primarily consists of collagen types IV and VI. However, during the development of fibrosis, the ECM is replaced by fibrillary collagens, such as collagen types I and III, along with fibronectin. This change leads to the capillarization of the sinusoids.^14^ When fibrosis becomes established and chronic liver disease progresses to cirrhosis, significant structural changes occur, including extensive capillarization of the liver sinusoids and the formation of intrahepatic vascular shunts. These changes are accompanied by functional abnormalities, particularly endothelial dysfunction. This dysfunction arises from a reduction in the endothelial synthesis of vasodilators, such as nitric oxide (NO), and an increase in the secretion of vasoconstrictors, such as thromboxane A2 and endothelin.^28,29^ These structural and functional changes lead to the development of portal hypertension, which is the major complication associated with liver cirrhosis. This condition, in turn, contributes to other significant complications of cirrhosis, including ascites, variceal bleeding, hepatic encephalopathy, and renal failure.^30,31^

Among the various growth factors, TGF-β plays a central role in the development of liver fibrosis.^32^ Liver macrophages, including Kupffer cells, are the primary producers of TGF-β, although HSCs can also secrete this growth factor. TGF-β binds to the type II TGF-β receptor, which then activates the type I TGF-β receptor. This process leads to the activation of both Smad-dependent and Smad-independent signaling pathways. In HSCs, TGF-β activates Smad2 and Smad3, which stimulates the synthesis of ECM proteins, such as type I and type II collagen, while also inhibiting their degradation. Overall, TGF-β plays a key role in the fibrogenesis process through various mechanisms.^32^

**Fig. 1:**
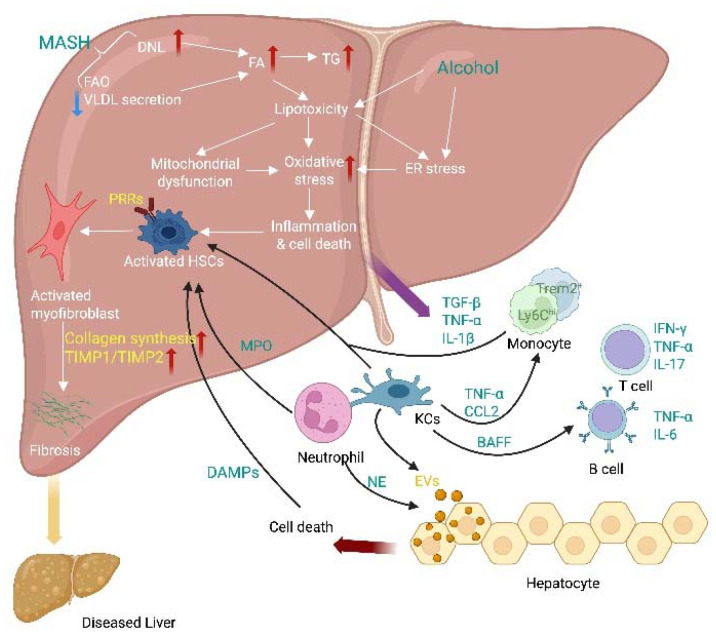
Schematic summary of the pathogenesis of liver inflammation and fibrosis. Innate immune responses involved in metabolic dysfunction and alcoholic liver disease include activation of resident Kupffer cells and recruitment of leukocytes (*e.g.*, neutrophils, monocytes) to the liver. Lymphocyte-mediated adaptive immunity is an additional factor promoting liver inflammation. EVs act as drivers of inflammation in liver inflammation, activating immune cells and HSC. Red arrows indicate upregulation and blue arrows indicate downregulation; DNL (*de novo* lipogenesis), FAO (fatty acid oxidation), VLDL (very low-density lipoprotein), PRRs (pattern recognition receptors), MPO (myeloperoxidase), DAMPs (damage-associated molecular patterns), NE (neutrophil elastase), BAFF (B cell-activating factor), EVs (extracellular vesicles), CCL2 (C–C motif ligand 2).

Fig. 1 illustrates the typical pathway for liver inflammation and subsequent fibrosis due to excessive alcohol consumption and metabolic dysfunction. These factors can cause increased lipid synthesis and uptake in the liver, which, when it surpasses lipid oxidation and excretion, leads to lipid accumulation and lipotoxicity. The latter processes trigger an inflammatory response, cell death, and eventually fibrosis.

## 3. Types of biomimetic coatings

CM NPs have emerged as an attractive tool in nanomedicine due to their unique properties, such as immune system evasion, long blood circulation time, and cell targeting.^22^ The cell performs a wide range of functions including interfacing with its surrounding environment. The natural properties of cell membranes can be utilized by coating NPs, thereby conferring upon the latter the biointerfacing functionalities inherent within the cell membranes.

RBC-derived membranes have distinct surface markers like CD47 and glycans, which play a key role in reduced phagocytosis of NPs by macrophages, resulting in prolonged circulation time. Platelet membranes also express CD47 surface markers, and have unique representation of glycoprotein and P-selectin, providing innate immune escape along with its natural ability to target inflammation. Whereas in cancer cell membranes, the presence of adhesion molecules and integrins on their surface confer homologous recognition. In the case of HCC, CD 147 is the key target molecule for homologous targeting. The macrophage membrane can target inflammation, neutralize inflammatory factors, and block inflammatory response, inheriting the source macrophage property.^33^ Therefore, depending on the desired application, the type of cell membrane to be used can be chosen. This technique gives rise to NPs that are facile, highly generalizable, and have the potential to greatly augment the potency and safety of existing nanocarriers. Furthermore, the coating of NPs with natural membranes enables additional applications for the formulation including dual functionality with properties similar to that of the natural membrane, while having a synthetic core, preservation of surface markers, and prolonged *in vivo* circulation.^23^ Various techniques are utilized for coating NPs derived from different cell types, such as extrusion, sonication, and self-assembly techniques, which allows integration of the biomimetic component in the final formulation, reducing the risk of an immune response and enhancing the stability of the NPs in the blood.^24^ Furthermore, different techniques are utilized for the functionalization of CM NPs. These techniques involve chemical modification, genetic engineering, and external stimuli. Chemical modification ensures the accurate control over the type and density of functional moieties on CM NPs, allowing customized modifications with desired therapeutic requirements. Genetic engineering combines the unique properties of both cell membranes and NPs to generate a versatile platform for various applications, including drug delivery and therapeutics. This technique can be used to modify the membrane before they are fused with NPs, which can be done by introducing specific genes or genetic modifications into the cells from which the membrane is derived.^25^ These modifications confer additional functionalities to the resulting CM NPs. External stimuli on the other hand involves the overexpression of specific membrane proteins in the cell membrane in response to external stimuli. This approach allows for the incorporation of additional functionalities into the CM NPs, thus enhancing their therapeutic potential and enabling targeted drug delivery. When used, the external stimuli cause an upregulation of membrane proteins, which confers the desired functionalities on the NPs, such as targeting ligands or receptors.^24^ There are different types of CMs used in coating NPs. These include red blood cell (RBC)-derived membrane-coated NPs, immune cell-derived membrane-coated NPs (macrophage-derived membrane-coated NPs, T-cell-derived membrane-coated NPs, NK cell-derived membrane-coated NPs, and dendritic cell-derived membrane-coated NPs), cancer cell-derived membrane-coated NPs, and platelet-derived membrane-coated NPs.^24^

Platelets carry out a significant physiological function in the body. They function primarily to maintain hemostasis and can be activated to undergo conformational changes from a disc shape to spiky spheres due to the influx of calcium ions.^26^ Once activated, platelets release their storage contents such as chemokine factors, glycoproteins, adhesive proteins, coagulation factors to promote an increase in the number of adhesion receptors and clotting proteins which leads to clumping and plug formation at the site of cell damage. As a membrane coating on NPs, they mimic the natural platelets and enhance the targeted delivery of the NPs, through surface modifications with ligands and biomarkers. They serve as excellent immunocompatible alternatives for the controlled and targeted delivery of biomolecules.^33,34^ Xie and his group demonstrated the biomedical applications of a platelet–neutrophil hybrid membrane-bound nanoformulation (PNM) for the treatment of MASH. They prepared gelatin NPs, co-loaded with pioglitazone and vitamin E within the hybrid membranes, and reported the enhanced immune evading ability of PNM due to the surface adhesion molecules present on them. They further demonstrated the high expression of matrix metalloproteinase-9 (MMP-9) at the MASH sites which facilitated the degradation of gelatin NPs to release of vitamin E and pioglitazone for drug treatment.^35^

**Fig. 2:**
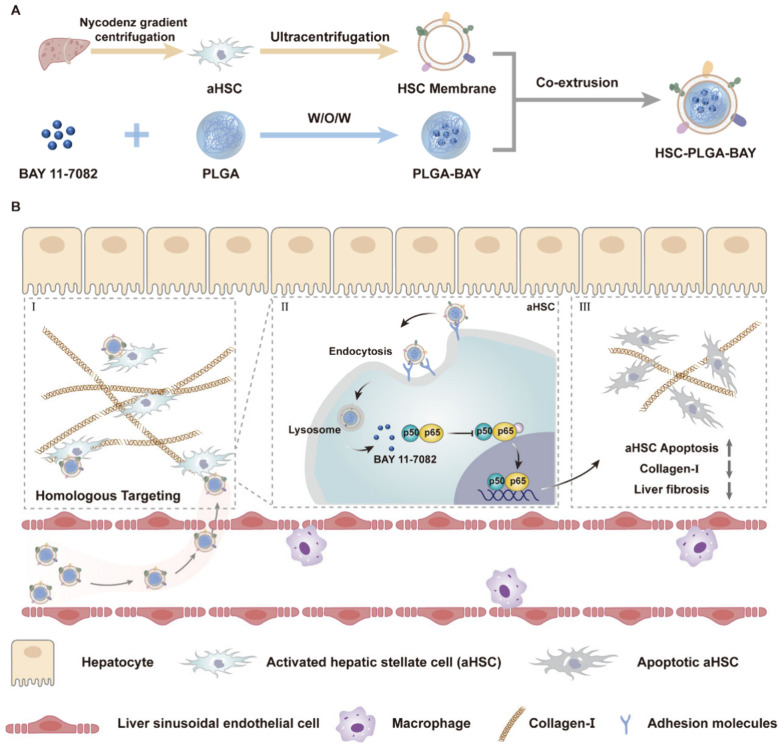
Schematic illustration of (A) the preparation of hepatic stellate cell coated polymeric NPs encapsulated with NF-κB inhibitor and (B) its anti-fibrosis mechanism. BAY 11-7082 encapsulated inside PLGA NP with HSC coating increased apoptosis of aHSCs, enhancing the apoptosis of aHSCs *via* inhibition of NF-κB pathway, ultimately causing a reduction in collagen production. Reprinted with permission from ref. 121. Copyright © 2024 American Chemical Society.

NF-κB plays a key role in regulating HSC activation by promoting cell survival during liver fibrosis. Within the aHSCs, the dysregulated NF-κB signals lead to prolonged cell survival and apoptosis resistance.^29^ Recently, Cheng and his coworkers developed a biomimetic nanosystem that comprises HSC membranes coated onto polymeric poly lactic-*co*-glycolic acid (PLGA) NPs encapsulated with an NF-κB inhibitor BAY 11-7082.^121^ This inhibitor of NF-κB been known to hinder the development and progression of liver fibrosis by inducing apoptosis in aHSCs. The HSC-coated PLGA NPs of size 108 nm showed homologous targeting towards aHSCs and demonstrated efficient treatment by increased apoptosis of aHSCs *via* inhibition of NF-κB pathway, ultimately causing a reduction in collagen production, promising to be an effective treatment modality for liver fibrosis (Fig. 2).

In another study by Xiao and his group, a cancer cell membrane coating was used to camouflage a metal–organic framework for hepatocellular carcinoma.^122^ The core comprised of pH-sensitive zinc imidazole framework NPs doped with ferrous ions and further encapsulated with dihydroartemisinin. The ferrous ion reacted with dihydroartemisinin under acidic pH and resulted in DNA damage and apoptosis by the generation of carbon-centered radicals. The homologous binding abilities of cancer cell membranes prevented the nanosystem from immune clearance and increase tumor accumulation in *in vitro* and *in vivo* systems.

## 4. Cargoes delivered by biomimetic NPs and their application

### 4.1. Chemotherapeutic drug delivery

Despite being a conventional treatment option for liver cancer, chemotherapy often encounters challenges due to multidrug resistance (MDR). MDR arises from various complex mechanisms, including abnormal expression of topoisomerase regulated by apoptosis-related genes, as well as the overexpression of P-glycoprotein (P-gp).^37–39^ Key strategy to overcome cancer MDR is through combination therapy by utilizing NPs for targeted delivery to specific subcellular organelles, and implementing multimodal combination therapy.^36,40,41^ The overexpressed P-gp levels in resistant cancer cells are particularly crucial, as they efflux chemotherapeutic agents out of the cells. Inhibiting this overexpressed P-gp is a direct approach to overcoming cancer MDR.^42^ Xu *et al*. reported a long-circulating liposome with two chemotherapeutics doxorubicin (Dox) and Schizandrin for liver cancer and reversing MDR with a reversion index of 30.28.^43^ The liposomal formulation with DSPE-PEG 2000 provided prolonged circulation. Schizandrin acted as an anticancer drug as well as a multidrug reversal agent and also provided a synergistic anti-cancer effect with Dox.^43^ In another study by Zhao *et al*., Dox and curcumin were co-delivered using lipid NPs for liver cancer chemotherapy. Curcumin acts as a chemosensitizer that suppresses the P-gp expression and thereby reverses MDR, while Dox is an anthracycline antibiotic that can be used in liver cancer treatment; however, due to its cardiotoxicity and poor therapeutic window, its clinical application is limited.^44^ These limitations can be overcome by delivering Dox and curcumin using lipid NPs for enhanced anticancer activity.^44^ ATRA is a Golgi-disturbing agent that transforms the Golgi apparatus into a diffuse vacuolar aggregate thereby increasing the toxicity of certain immunotoxins entering cells by receptor-mediated endocytosis.^45^ It has been shown to reduce fibrosis associated with liver cancer, and a combination of ATRA and Dox-loaded lipid NPs against liver cancer showed greater antitumor efficacy.^46^ Luo and his group used chondroitin sulfate lipid NPs loaded with Dox and retinoic acid for liver cancer management. Chondroitin sulfate interacted with *N*-acetylgalactosaminyltransferases for targeting the Golgi apparatus and was efficiently internalized by liver cells through the CD44 mechanism. The retinoic acid inhibited ECM protein production in the liver by destroying the Golgi structure while the death of hepatoma cells and HSCs was triggered by Dox by disrupting their DNA function. Confocal microscopy confirmed that coumarin 6 dye-loaded chondroitin sulfate NPs localized to the Golgi in hepatoma cells and HSCs following uptake, while the other treatment groups (coumarin 6 solution and coumarin 6-containing NPs without chondroitin sulfate) did not. The chondroitin sulfate NPs were also retained longer (atleast 6 hours) and showed greater anti-tumor effects than the other treatments in primary liver cancer mouse models. In another study by Rui *et al*., a recombinant high-density lipoprotein NP was developed to encapsulate and co-deliver Paclitaxel and Dox for combination chemotherapy to treat hepatocellular carcinoma.^47^ The recombinant high-density lipoprotein was formed by self-assembly of apolipoprotein A1, which is the major functional protein in natural high-density lipoprotein (HDL) and it possesses a specific affinity to HDL receptor scavenger receptor class B type 1, expressed on malignant cells and deliver the cargo to the cytoplasm directly. The co-loaded formulation showed higher tumor growth inhibition compared to free drug cocktails, reducing tumor volume by over 60% at optimal drug ratios (1 : 2.3 and 1 : 1.1, Ptx to DOX). The synergistic effect of Dox and Ptx was confirmed by checking the combination index, which was below 1, for the dual-loaded HDL NPs, making them an efficient delivery of multiple chemotherapeutics.^47^

### 4.2. Gene delivery

RNA interference (RNAi) is a process that specifically inhibits the expression of genes with matching sequences, leading to gene silencing.^48–50^ siRNA drugs exhibit high efficiency and specificity, which offers promising new approaches for gene therapy in hepatic fibrosis. The molecular pathology of hepatic fibrosis makes it feasible to develop siRNA drugs capable of reversing the condition.^51,52^ However, naked siRNA can be easily degraded *in vivo* due to the presence of serum enzymes, and high clearance, and often its silencing effect gets hindered by off-target effects. Nano-delivery systems based on cationic lipids have been widely studied for delivering negatively charged siRNA with increased transfection efficiency and entrapment. Jia *et al.* reported a cyclic oligopeptide-modified cationic nucleic acid-lipid NPs for targeted hepatic fibrosis therapy. The cyclic oligopeptide pPB has a strong binding affinity with platelet-derived growth factor receptor-β (PDGFR-β) which is overexpressed on activated HSCs and can be considered an effective targeting moiety for hepatic fibrosis.^53^ In another study, pPB peptide-modified HMGB1-siRNA-loaded nucleic acid-lipid NPs were prepared to treat liver cirrhosis. These NPs have dual antifibrotic and anti-inflammatory activities.^51^ The HMGB1-siRNA silenced the HMGB-1 gene, targeted HSCs through the pPB peptide, inhibited HSC proliferation, and promoted HSC apoptosis.^51^ Wang and his group reported transferrin-modified liposomes for the delivery of the acetylcholinesterase gene to the cytoplasm *via* transferrin receptor-mediated endocytosis. Acetylcholinesterase is identified as a key marker in liver cancer. It can degrade acetylcholine and reduce the malignancy risk, and it can be used as a gene therapy for liver cancer.^54^ Transferrin is overexpressed in tumors and is responsible for transporting iron into cells. The targeting ability was confirmed with fluorescent microscopy and flow cytometry, which showed an increased uptake in Transferrin-modified NPs on hepatocarcinoma cells compared to normal liver cells. The Transferrin-modified NPs loaded with acetylcholinesterase gene (Tf-PL/AChE) significantly inhibited the proliferation of liver cancer *in vivo*. In *in vitro*, Tf-PL/AChE demonstrated higher transfection efficiency than the commercial transfection agent Lipo 2000, and more significant cytotoxicity against SMMC-7721 cells (IC50 values of 4.25 μg mL^−1^ at 48 hours and 3.45 μg mL^−1^ at 7 hours), when compared to unencapsulated (free gene and unmodified NPs). *In vivo*, Tf-PL/AChE achieved a tumor inhibition rate of 77.47% in xenograft models, significantly reducing tumor volume (517.14 ± 112.63 mm^3^) and showing prolonged tumor retention. The system had a high % encapsulation efficiency of 94.3%, induced apoptosis, and arrested cell cycles, highlighting Tf-PL/AChE as a promising non-viral gene delivery platform for targeted liver cancer therapy.^54^

### 4.3. Anti-fibrotic drug delivery

Pirfenidone, Elafibranor and Obeticholic acid are few of the FDA-approved hepatic fibrosis drugs which are still in clinical trials. The poor targeting ability and short half-life of anti-liver fibrotic drugs make their clinical use difficult. Using biomimetic nanomaterials including liposomes and membrane-coated NPs enables the targeted delivery of drugs. As described previously, activated HSCs are the key source of ECM deposition and therefore the main triggers in liver fibrosis. Designing a delivery system that targets aHSCs helps achieve specific inhibition of aHSCs for liver fibrosis. Zhang and his group reported a hybrid biomimetic system by constructing bone marrow mesenchymal stem cells-derived exosomes and Vitamin A-modified liposome membranes for delivering an autophagy inhibitor hydroxychloroquine. aHSCs have high expression of retinol-binding protein and vitamin A can be used to target aHSCs *via* retinol-binding protein receptors. This aHSC-specific nano-delivery system showed enhanced drug uptake by selective targeting to aHSC but also reduced damage to other liver cells.^55^ Chimeric antigen receptors (CAR) T cells have the innate ability to eliminate tumor cells by specifically recognizing their tumor-associated antigen. In a report by Ma *et al*., the cell membrane of CAR T cells was used to construct a membrane-coated nanoplatform with IR 780-loaded mesoporous silica NPs for photothermal therapy.^56^ CAR-T cells also have the property to target Glypican-3 in hepatocellular carcinoma and thus glypican-3 targeting CAR T cell membrane camouflaged NPs showed enhanced tumor-targeted therapy.

During fibrosis, the HSCs get activated and as a result, secrete excessive TGF-β affecting its signalling pathway. Peroxisome proliferator-activated receptor-gamma regulates the TGF-β1/Smad signalling pathway. Baicalin targets peroxisome proliferator-activated receptor-gamma and regulates their immune-mediated chronic inflammatory signaling inhibits PDGF-BB-induced HSC activation and ameliorates LPS-induced HSC migration, making Baicalin an effective therapeutic agent.^57^ Sun *et al*. reported the use of a neutrophil membrane that endows the neutrophil-like properties to the NPs, and sequential delivery of atorvastatin, ambrisentan, and Baicalin for the treatment of liver fibrosis. Firstly, atorvastatin and ambrisentan-loaded neutrophil membrane nanosystems specifically targeted the inflammatory part of the liver and normalized the capillarized liver sinusoidal endothelial cells (LSECs) by the synergistic effect of drugs. Consequently, the second nano system comprising vitamin A-modified liposome-neutrophil membrane NPs with Baicalin was delivered to the aHSCs and inhibited HSC activation making it ideal for the treatment of liver fibrosis.^58^

### 4.4. Image-guided ultrasound/photothermal therapy

**Fig. 3:**
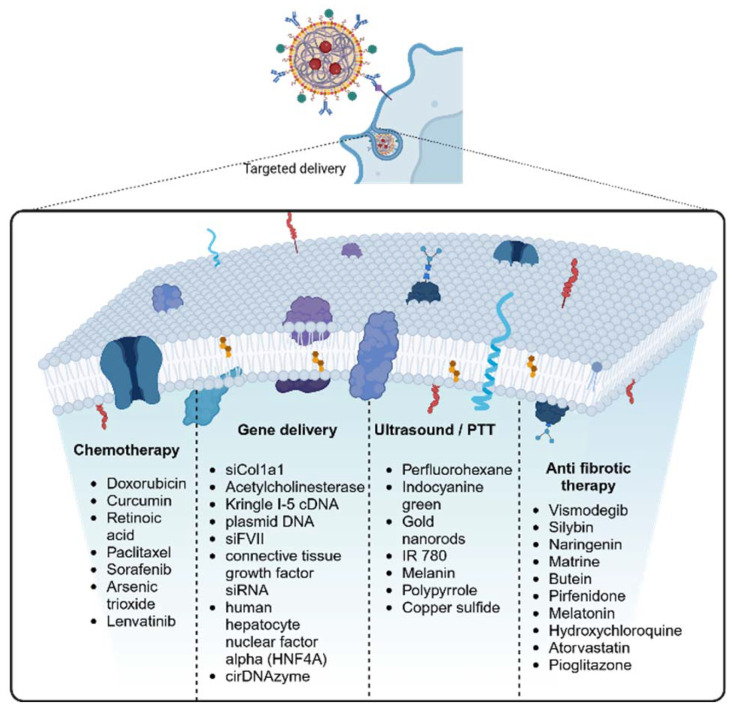
Schematic diagram of biomimetic NPs targeted delivery and the different types of cargo delivered using these biomimetic NPs.

Photoacoustic tomography (PAT) with its high spatial resolution and deep penetration ability provides a novel opportunity for early diagnosis which enhances tumor detection, diagnosis, and delineation. Indocyanine green (ICG) is an FDA-approved photoacoustic and fluorescent dye, commonly used in clinical practice, which could detect new nodules and identify more than 95% of lesions in patients, thereby improving the precision of resection. However, the photoacoustic signal of ICG decreases in human liver specimens and is not detected in orthotopic liver cancer in mice due to fluorescence quenching, which urges the need for a suitable probe with fluorescence and enhanced photoacoustic ability. Guan *et al*. fabricated one such dual-modality probe that includes a liposomal formulation loaded with ICG for NIR fluorescence and gold nanorods for effective photoacoustic imaging.^59^ The fabricated NPs formed a core–shell structure with ICG-loaded gold nanorods forming the core, and liposomal layer forming the shell, with a particle size of around 78 nm. The stable absorption at 795 nm increased the PAT signal, which was confirmed with the *in vitro* and *in vivo* PAT and bioluminescence imaging.^59^ In another study by Wu and his group, polypyrrole NPs loaded with Dox were coated with platelet membranes for combined chemo-photothermal therapy of hepatocellular carcinoma. Polypyrrole NPs when laser irradiated generate photothermal conversion thereby increasing the triggered release of Dox from the core.^34^ The platelet membranes showed immune-evasion property and tumor-targeting properties due to their innate membrane property, in *in vitro* and *in vivo* orthotopic tumor models. The photothermal properties of polypyrrole, when irradiated with an 808 nm laser caused a temperature increase to 50 °C *in vitro*, and led to the lowest tumor weight *in vivo* making this combination therapy effective in achieving tumor ablation.^34^ Ji and his group developed a hollow copper sulfide NP loaded with sorafenib for combined phot-chemotherapy of hepatocellular carcinoma. They further decorated the hollow NPs with a hybrid membrane of macrophage and cancer cells which provided the nano system with innate immune escape and tumor-targeting properties. Copper sulfide NPs showed synergistic photothermal conversion ability upon laser irradiation.^60^Fig. 3 shows the list of different drugs delivered using biomimetic NPs for different applications.

## 5. Materials used for biomimetic NP development for liver disease targeting

### 5.1. Biomimetic NPs for liver fibrosis

Different mechanisms and approaches are currently being employed in the targeted biomimetic NP-based delivery of antifibrotic drugs to the liver to reverse hepatic fibrosis. These include the formulation of liposomes and the use of biomimetic/cell membrane drug delivery systems.

#### 5.1.1 Liposomes

Liposomes have been extensively studied as drug carriers for treating liver fibrosis. Table 1 lists some of the receptors and ligands explored recently for liver fibrosis treatment. Recently, Wang *et al.* developed albumin self-modified liposomes to deliver naringenin – an anti-fibrotic agent and Smad3 protein inhibitor, to activated HSCs in liver fibrosis. The albumin can target cysteine (SPARC) receptor upregulated in activated HSCs during fibrosis. More than 1.5 times greater uptake of the albumin-containing liposomes than the liposomes without albumin was observed in activated HSCs *in vitro.* The formulation also had greater toxicity towards HSCs than naringenin alone and naringenin-containing liposomes without albumin, further confirming their specificity towards HSCs.^61^

**Table 1:** List of some of the receptors and ligands used recently for liver fibrosis therapy

Receptor	Ligands
Secreted protein acidic and rich in cysteine (SPARC) receptor	Albumin^61^
CD44	Hyaluronic acid^64^
Platelet-derived growth factor receptor-β (PDGFR-β)	pPB peptide^53^
Sigma receptor	Anisamide^49^
Asialoglycoprotein receptors	*N*-Acetyl-d galactosamine^65^
Chemokine receptor 4 (CXCR4)	AMD3100^66^
Retinoic acid receptors	Vitamin A^67^
Collagen type VI receptor	Cyclic RGD peptide^68^
Insulin-like growth factor II receptor or mannose-6-phosphate receptor	Mannose-6-phosphate^69^
Angiotensin II type 1 receptors	Losartan^70^

Several other different types of phospholipids and surface modifications have been explored by research groups in the development of liposomes to enhance the delivery of proteins and peptides to fibrotic cells.^62^ Among phospholipids, 1,2-distearoyl-*sn-glycero*-3-phosphocholine (DSPC) and 1,2-dioleoyl-*sn-glycero*-3-phosphoethanolamine (DOPE) are the most utilized as fusion-promoting phospholipids to facilitate cellular entry. A study conducted by Yuan *et al.* utilized DSPC to formulate curcumin-loaded liposomes, conjugated with a choline polymer for effective delivery to fibrotic cells. They observed that the formulation improved the stability of curcumin and significantly decreased the expression of α-SMA in aHSCs. *In vivo* studies in carbon tetrachloride (CCl_4_)-induced mice model showed alleviation of liver injury, and collagen deposition, which facilitated a reversal of liver fibrosis.^63^

Another study by Zhang *et al.*, involved developing a hybrid biomimetic drug delivery system using a combination of liposomes and exosomes derived from bone marrow mesenchymal stem cells and modified by Vitamin A for targeted delivery to aHSCs. In addition, hydroxychloroquine which is an autophagy inhibitor was encapsulated for synergistic anti-fibrotic effects.^55^ Depletion of Vitamin A leads to the progression of liver fibrosis. About 80% of Vitamin A is stored as retinyl esters in healthy liver cells, primarily the quiescent HSCs. These reserves are depleted during liver injury, activating HSCs and decreasing liver retinyl esters and retinol concentrations. The Vitamin A depletion and HSC activation in turn initiates a series of cascading events that leads to ECM deposition.^71^ Another study developed hybrid liposomes, functionalized with Vitamin A, in a fluorinated peptide/lipid conjugate to co-deliver Sorafenib and siRNA against heat shock protein 47 (HSP47). They demonstrated high loading efficiencies and sustained release of the drugs while improving aHSC targeted delivery due to the binding affinity of Vitamin A to retinol-binding protein receptors on HSCs. The NPs also significantly reduced ECM deposition by HSC-T6 cells, which was confirmed by the decreased levels of liver fibrosis-associated genes HSP47, TIMP-1, and collagen I. The enhanced breakdown and reduced synthesis of collagen restored liver function in liver fibrosis mouse models, shown by low levels of serum liver transferases, collagen accumulation, and reduced α-SMA and CD-31 expression, thereby relieving liver fibrosis.^72^

Another interesting study on aHSC-specific drug delivery was done by Lee *et al.*, who reported the development of NPs that are activated by fibroblast activation protein (FAP) overexpressed by aHSCs. The formulation consisted of promelittin peptide conjugated to polyethylene glycol (PEG)- and maleimide-functionalized liposomes, to obtain promelittin-modified liposomes. The promelittin was cleaved in the presence of FAP to release the anti-fibrotic peptide melittin in a site-specific manner at the aHSCs, for increased therapeutic efficacy. The formulation decreased aHSC proliferation, induced significant aHSC death in three different mouse models of liver fibrosis, *i.e.*, the CCL_4_-induced, high-fat diet-induced and bile duct ligation models, and improved the overall survival rate particularly in the latter mouse model.^73^ Several nucleic acid-containing lipid-based drug delivery systems are currently under clinical trials. One such formulation is ND-L02-s0201, which encapsulates siRNA that targets HSP47. Developed by Bristol-Myers Squibb, the formulation has been evaluated for its pharmacokinetic and safety profiles in Phase 1b/2 clinical trials for the treatment of liver fibrosis.^74,75^ As mentioned earlier, the progression of liver fibrogenesis, and aHSC is controlled by numerous profibrotic cytokines, and the fibrosis progression can be mitigated using RNAi to downregulate the cytokines.^76^ A lipid-based NP consisting of a cationic amphiphile was formulated to conjugate a highly branched siRNA with a helper lipid (cholesterol-polyethylene glycol-vitamin-A) for targeted HSC delivery. The formulation showed a decreased accumulation of collagen in CCl_4_-treated mouse models and demonstrated enhanced gene-binding ability, transfection efficiency, and improved delivery of siRNA to aHSC.^77^ Another notable formulation was reported by Li *et al.*; to enhance the uptake of Sorafenib at low doses, they conjugated the CREKA (Cys-Arg-Glu-Lys-Ala) peptide composed of five amino acids (which has a high affinity for fibronectin, an ECM protein produced by HSCs), to liposomes.^78^ They observed that the CREKA-liposomes not only facilitated the delivery of Sorafenib to aHSCs, but also promoted the uptake of the drug by the human HSC cell line LX2, with selective accumulation in *in vivo* carbon tetrachloride (CCl_4_)-induced fibrotic mice liver through the recognition of fibronectin. Furthermore, *in vivo* studies showed that the CREKA-modified liposomes reduced liver fibrosis by inhibiting angiogenesis in mice.^69,79^

Integrins are a large family of heterodimeric cell surface receptors, made of α and β subunits that respond to ECM by regulating cell surface attachment.^80^ These complexes have varying degrees of affinity to extracellular ligands, and regulate cell growth, proliferation, migration, signaling, and cytokine activation and release and thus play a significant role in cell proliferation, migration, apoptosis, tissue repair, and all processes crucial to inflammation, infection, and angiogenesis.^81^ Researchers also conjugated the peptide Cyclo [Arg-Gly-Asp-DTyr-Lys] (cRGDyK), which binds to integrin αvβ3, to liposomes for the delivery of vismodegib, a hedgehog inhibitor, to aHSCs. These liposomes were selectively taken up by aHSCs *in vitro* and *in vivo*, unlike qHSC or other liver cells.^82^

Clodronate is a hydrophilic molecule that can be encapsulated within liposomes. Free clodronate does not easily cross the cell membrane and is rapidly cleared (*i.e.* within minutes) from circulation by the renal system. However, when entrapped in a liposome, the clodronate-liposome is ingested by macrophages, where the liposomes are digested by lysosomal phospholipases, leaving the clodronate undigested within the macrophage. The more liposomes are ingested by the macrophage, the more the accumulation of clodronate within the cells which eventually causes apoptosis of the macrophage.^83^ This mechanism was utilized by Ji *et al.* to effectively co-deliver Nintedanib and clodronate using an exosome–liposome hybrid drug delivery system, for liver fibrosis therapy. The clodronate in the liposomes enabled Kupffer cell inhibition, leading to decreased non-specific phagocytosis of the particles and reduced production of inflammatory cytokines. This dual inhibitory effect on Kupffer cells enhanced Nintedanib delivery to fibroblasts in CCl_4_-induced fibrotic mice livers by leveraging the homing capability of the homologous EVs in the formulation. The group thus achieved improved anti-fibrotic therapeutic outcomes with their final formulation, when compared to formulations without clodronate.^84^ Similarly, a relaxin-encapsulated liposome conjugated with aminoethyl anisamide was developed to preferentially target aHSCs in the fibrotic liver *via* the sigma-1 receptor. Relaxin is an endogenous peptide hormone that relieves fibrosis by reversing aHSCs. The encapsulation of relaxin in lipid NPs led to a 20-fold increase in relaxin in the liver but not in other organs, and enhanced its antifibrotic effects in CCl_4_-induced liver fibrosis mouse models.^85^

Natural products have also been encapsulated within liposomes for liver fibrosis applications. Oxymatrine (OM), an alkaloid extracted from the medicinal plant *Sophora alopecuraides* L, has gained popularity for its inhibitory effect on the replication of hepatitis B and C viruses *in vitro*. Preclinical and clinical data revealed beneficial effects on the progression of liver fibrosis. The efficacy of OM-loaded liposomes, conjugated to a peptide was investigated by Chai *et al.*^86^ Their results revealed that OM delivered by the liposomes decreased hepatic fibrosis in CCl_4_-induced rats, shown by a decrease in serum alkaline phosphatase, in addition to a decrease in liver injury and suppression of the fibrosis-related genes. Furthermore, the formulation improved the targeting of OM to HSCs resulting in decreased cell viability, triggered HSCs death *in vitro*, and enhanced its therapeutic effects. It was noted that fluorescent-labeled liposomes preferentially accumulated within hepatic phagocytes, and in T cells. Dexamethasone loaded within the liposomes decreased T cells in the blood and liver and induced anti-inflammatory polarization of macrophages more efficiently than the free Dexamethasone. Reduced liver damage and necrosis and reduced collagen content were also observed in mouse models with CCl_4_-induced liver injury.^87^ Imatinib, a medication for the treatment of cancers impedes PDGF and TGF-β pathways, making it applicable in treating liver fibrosis. Although low concentration at the site of action and off-target toxicities characterizes imatinib therapy, encapsulating it within liposomes and conjugating them with Vitamin A can improve imatinib therapy by specifically targeting HSCs. This formulation demonstrated high encapsulation efficiency, with a 13.5-fold uptake in the liver compared to free imatinib, and less uptake by other organs, leading to reduced off-target effects.^88^

**Fig. 4:**
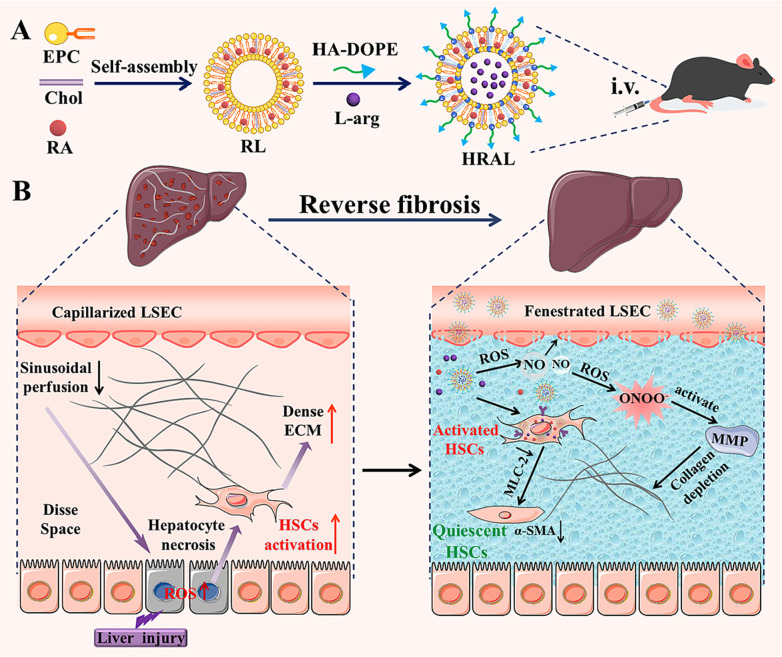
Schematic illustration of (A) HA-modified liposomes to co-deliver ATRA and l-arginine (HRAL); (B) the formulation targets HSCs to normalize pathological barriers and help in reversing fibrosis. Reprinted from Wang *et al*.,^64^ under a creative Commons Attribution (CC BY) license (https://creativecommons.org/licenses/by/4.0/).

Wang and his coworkers used Hyaluronic acid (HA) modified liposomes for co-delivery of ATRA and l-arginine to reverse fibrosis (Fig. 4). The HA binds to CD44 receptor overexpressed on aHSCs, which led to greater uptake by HSCs than normal hepatocytes. In the fibrotic environment, l-arginine, an endogenous NO donor, is oxidized by ROS or catalyzed by endothelial nitric oxide synthase to generate NO, which repairs the fenestrae of impaired LSECs, allowing the liposomes to cross the hepatic sinusoidal barrier. The subsequent oxidation of NO activates MMPs to break the ECM barrier, facilitating the internalization of the liposomes. After being taken up by aHSCs, ATRA is released, which suppresses HSC activation, thereby reversing fibrosis.^64^

#### 5.1.2. Cell-membrane-coated NPs

There are multiple reports on the use of NPs coated with different cell membranes for liver fibrosis treatment. Biomimetic NPs based on autologous fibroblasts are customized as baits to neutralize multiple fibroblast-targeted cytokines. By fusing the skin fibroblast membrane onto PLGA cores, these NPs, termed fibroblast membrane-camouflaged NPs, are shown to effectively scavenge various profibrotic cytokines, including TGF-β, interleukin (IL)-11, IL-13, and IL-17, thereby modulating the profibrotic microenvironment.^89^

Another study conducted by Bai *et al.* utilized platelet membranes and HSC membranes to coat melatonin for delivery to liver fibrotic cells in mice models. Although melatonin is effective in relieving liver fibrosis by inhibiting the endoplasmic reticulum stress receptor, it is not readily soluble and has low bioavailability when taken orally. The membrane coating improved the efficacy and targeted delivery of melatonin in liver fibrosis and showed good safety profiles.^90^ Similarly, macrophage membrane-coated polydopamine NPs effectively quenched pro-inflammatory cytokines (TNF-α, IL-6, and IL-1β) expressed in liver fibrosis, with improved NP uptake, targeting, anti-inflammatory and antioxidant effects compared to the free macrophage membrane and polydopamine.^91^

Metal–organic frameworks (MOF) have also been utilized as a core in biomimetic NPs for the encapsulation and targeted delivery of macromolecules, and chemotherapeutic drugs.^92,93^ Biomimetic Zinc imidazole frameworks (ZIF-8) NPs exhibit a flexible and promising strategy for treating HCC, combining targeted drug delivery, controlled release, and the potential for multifaceted therapy. A study conducted by Ni *et al.* evaluated the targeted delivery capabilities and biocompatibility of a ZIF-8 nanoformulation that encapsulated ICG and doxorubicin and coated with macrophage membrane to treat HCC. The NPs were effectively internalized within the cancer cells, leading to inflammatory cell necrosis. Despite being regarded as an innovative advancement in drug delivery, there is limited literature on the application of these biomimetic MOF-based biocomposites for delivering biomolecules to fibrotic liver cells, presenting an opportunity for further exploration.^94^ Another study by Qin and his group presents the fabrication of ZIF-8 lipid NPs loaded with pirfenidone and Vitamin A for the treatment of liver fibrosis. The ZIF-8 lipid NPs construct showed a particle size of 84.3 nm and a loading efficiency of up to 54%. Additionally, the modified Vitamin A had a high binding affinity for retinol-binding protein receptors overexpressed on the surface of HSCs, with enhanced uptake into fibrotic cells.^95^Table 2 shows a list of different types of membrane coatings and core NPs for liver fibrosis.

**Table 2:** List of different types of membrane coatings and core NPs for liver fibrosis

Biomimetic coating	Core NPs	Encapsulated biomolecule	Target	Outcome	Ref.
Mitochondrial membrane coating	PLGA		ABT-263, (B-cell lymphoma protein 2 (Bcl-2) inhibitor)	Increase in NPs from 103 to 128 nm	96
				Change in zeta potential from −42.2 to −25.0 mV due to the charge screening by the OMM coating	
				Membrane thickness of 9.0 nm similar to 7.5 nm for outer mitochondrial membrane (OMM)	
				Coated NPs exhibit similar protein profiles with OMM lysate, indicating the preservation of membrane proteins.	
				Control of the particle shape and elasticity enhanced accumulation within the liver	
Red blood cell membrane	Ethyl hydroxyethyl cellulose		TGF-β	Coated NPs enhanced liver cell proliferation *in vitro*, with reduced (<20%) internalization by macrophages.	97
	PLGA	Mesenchymal stem cells	Immortalized human adult liver epithelial cells	Prolonged circulation time	98
			Rat Hepatic Stellate Cell Line (HSC-T6)		
	Mesoporous silica composite	Silibinin	Mouse lung cells	Consistent spherical NPs were obtained	99
Mesenchymal stromal cell membrane (MSCM)	PLGA	Cryptotanshinone (CPT)	Liver fibrosis	Particle size increase from 183.9 to 204.3 nm	55
			Human fetal hepatocyte cell line (L-02) in acute liver injury	PDI (from 0.225 to 0.270)	
				Zeta potential (from −21.7 mV to −9.6), approx. like that of MSCM (−10.5 mV)	
Macrophage cell membrane	PLGA	Carvedilol	Murine AML12 hepatocytes	Coated NPs possessed negatively charged surface (−31.2 mV) like M2-type macrophage (−38.5 mV)	100
			RAW264.7 cells (Macrophage -like, Abelson leukemia virus-transformed cell line)	Macrophage Mannose Receptor (CD206), CC chemokine receptor 2 (CCR2), and tumor necrosis factor receptor 2 (TNFR2) were preserved in the coated NPs	
				Coated NPs had a sustained release of Carvedilol (<50%) compared to uncoated NPs (>97.4%) within 24 h	
				Coated NPs enhanced the biocompatibility in both AML12 and RAW264.7 cells	
				Treatment with coated NPs increased the anti-apoptotic protein level Bcl-2, but expression of pro-apoptotic proteins like cleaved caspase-3, cleaved poly(ADP-ribose) polymerase-1 (PARP-1) and Bax were downregulated compared to uncoated NPs	
Neutrophil membrane	Liposomes	Atorvastatin/amlisentan	Liver fibrosis	Normalization of LSECs through the elevation of tissue p-Akt protein and endothelial nitric oxide synthase following treatment with Atorvastatin/Amlisentan	58
		Vitamin A		Albumin-vitamin A coated NPs inhibited HSC activation by modulating the Peroxisome proliferator-activated receptor gamma (PPAR γ)/TGF-β1 and STAT1/Smad7 signaling pathways	

### 5.2. Biomimetic NPs for liver cancer

The use of biomimetic materials for targeted delivery of anti-cancer therapies to the liver can overcome current limitations in therapy including short drug half-life, non-specificity and drug resistance. We will now discuss ongoing research on the use of liposomes and cell membrane-coated NPs for targeted drug delivery to treat liver cancer, which has rapidly become one of the fastest-growing cancer types in the world.

#### 5.2.1. Liposomes

Several liposomal drug delivery systems have been developed and studied to target drugs specifically to liver cancer cells while minimizing adverse side effects. As an example, Haiwei Ye *et al.* prepared sorafenib nanoliposomes conjugated with VEGFR (vascular endothelial growth factor receptor) antibody for liver cancer therapy. Sorafenib inhibits several tyrosine kinases, such as VEGFR, which is involved in tumor angiogenesis and progression. These nanoliposomes demonstrated a 92.5% drug entrapment efficiency, effective targeting of VEGFR, and strong anti-cancer effects *in vivo* and *in vitro*. After 48 hours and 72 hours of incubation, the nanoliposomes showed approximately 30% and 18% more efficacy respectively compared to free sorafenib, which had a survival rate above 70% at the same concentration in Huh-7 cells. However, sorafenib only improves the survival rate of patients by three months, and some patients develop resistance due to metastasis.^101,102^ Alrashidi *et al.* found that resveratrol with sorafenib shows a synergistic effect when encapsulated in PEGylated liposomes. Resveratrol has anti-cancerous activity at multiple stages of tumor development and progression and minimizes toxicity to normal cells. PEGylated resveratrol with sorafenib liposomes inhibited tumor growth in BALB/c mice and had the lowest tumor volume (380.4 mm^3^) and weight gain (22.7%) compared to free resveratrol/sorafenib, resveratrol with sorafenib liposomes, and control groups. The formulation showcased adaptability to physiological conditions, sustained drug release, and superior stability over three months.^103^

In addition to commonly targeted receptors in cancer such as VEGFR, EGFR, and PDGFR, there are liver cell-specific surface receptors that can be leveraged to deliver therapies to specific cells that contribute to tumor growth and progression.^104^ An example is the asialoglycoprotein receptors (ASGPR), which are overexpressed in hepatocytes. Some studies have indicated that lipids can be modified with a lactose moiety to enhance the targeting of liposomes to liver cancer cells *via* a pathway that relies on ASGPR receptors. Zhou *et al.* reported the development of a novel lactosylated liposomal vehicle modified with Lac-DOPE (*N*-lactosyl-dioleoylphosphatidylethanolamine) that exhibited more robust tumor growth inhibition activity than liposomal-doxorubicin and free doxorubicin. These lactosylated liposomes showed enhanced uptake and higher toxicity in HepG2 cell lines when compared to non-targeted doxorubicin-containing liposomes. The formulation also increased tumor tissue accumulation through receptor-mediated endocytosis, and prolonged blood circulation time with a half-life of 8.73 hours compared to free doxorubicin which had a half-life of only 1.96 hours.^105^

Several studies have presented modified NPs with HA for targeted liver cancer treatment. HA holds a significant affinity for CD44 receptors which are generally overexpressed in cancerous cells.^106^ Sun *et al.* formulated a HA-modified liposome-based Icaritin delivery system for treating hepatocellular carcinoma.^106^ Icaritin influences key signaling factors, such as chemokine receptor type 4 (CXCR4), NF-κB, STAT3, and estrogen receptor alpha-36 (ERα36), while also modulating microRNAs, ROS, and sphingosine kinase-1.^107,108^ Additionally, Icaritin affects the receptor for advanced glycation end products – high mobility group box 1 (RAGE-HMGB1) pathway which significantly contributes to liver cancer progression by promoting tumor cell proliferation, invasion, and metastasis. When HMGB1 binds to the RAGE receptor on cancer cells, it activates inflammatory signaling cascades, resulting in increased tumor growth and aggressiveness.^109,110^ The HA acid-modified Icaritin liposomes showed superior cytotoxicity and tumor targeting in the CD44-expressing Huh-7 cell line with an IC50 of 34.2 μM, while unmodified Icaritin liposomes had an IC50 of >50 μM. IC50 values of both formulations were similar in HepG2 cells, which had a comparatively lower CD44 expression than Huh-7 cells. When compared to the control untreated group, the free Icaritin, Icaritin liposomes, and HA-modified Icaritin liposomes demonstrated tumor growth inhibition rates of 32.5%, 40.4%, and 63.4%, respectively *in vivo* in tumor-bearing nude mice, confirming the enhanced anti-tumor effects of the developed formulation.^106^

As the tumor grows, it continuously recruits myeloid cells that express folate receptors, so that they can bind to folate to support their own proliferation as well as promote tumor growth and proliferation; these folate receptors are attractive targets for NP delivery.^111^ Liu *et al.* developed a diacid metabolite of norcantharidin (DM-NCTD) loaded, folic acid (FA)-modified, polyethylene glycolated liposome system to enhance the targeting effect and antitumor potency of diacid metabolite of norcantharidin for HCC. Clinical studies have shown that the DM-NCTD is a promising inhibitor of protein phosphatase 1 (PP1) and protein phosphatase 2A (PP2A). The dysregulation of both PP1 and PP2A have been implicated in cancer development.^112^ FA has a high affinity, small size, and nontoxicity as a ligand. The FA-conjugated liposomes with a mean particle size of 200 nm and over 80% encapsulation efficiency demonstrated stronger cytotoxicity in H22 hepatoma cells with IC50 values of 95.3 μg mL^−1^ compared to 164 μg mL^−1^ for PEG liposomes without FA at 24 hours. Biodistribution studies in H22 tumor-bearing mice confirmed higher tumor-targeting efficacy with DM-NCTD/PEG liposome and DM-NCTD/FA-PEG liposome groups, with the values ranging from 12.81% and 24.44%, respectively, compared with DM-NCTD group (5.4%). Also, DM-NCTD/FA-PEG liposomes showed superior tumor inhibition and apoptosis induction with minimal off-target toxicity, highlighting their promise for HCC therapy.^112^

Dual-action drugs such as glycyrrhetinic acid (GA) has shown promising effects in liver cancer therapy due to its selective targeting ability and anticancer properties. GA targets liver cells by interacting with receptors overexpressed in HCC, specifically protein kinase C (PKC) and 11β-hydroxysteroid dehydrogenase type 1 (11β-HSD1). This selective binding improves drug delivery to liver cells and minimizes off-target effects.^113^ Dinh *et al.* synthesized Murrayafoline A (MuA) loaded glycyrrhetinic acid-modified liposomes, which showed improved targeting and cytotoxicity compared to non-targeted liposomes. The IC50 values in HepG2 cells were 7, 3.5 and 1.5 μM for MuA, MuA-loaded liposomes and MuA-loaded glycyrrhetinic acid-modified liposomes respectively. In contrast, the IC50 of the liposomes upon treating endothelial cells was 15 μM.^113^

Uptake of the liposomes by HepG2 cells and endothelial cells was 8.83 ± 0.97 ng 10^−5^ cells and 3.62 ± 0.61 ng 10^−5^ cells respectively. These studies confirm the specificity of the formulation for liver cancer cells. Tests on a 3D HepG2 cancer spheroid model demonstrated that GA-modified liposomes provided enhanced cell penetration and sustained MuA release, resulting in higher cell death rates than other formulations.^113^ A list of different liposomal formulations with their targeting ligand and receptor of interest for liver cancer treatment IS shown in Table 3.

**Table 3:** List of different liposomal formulations with their targeting ligand and receptor of interest for liver cancer treatment

Targeting ligand	Target receptor	Formulation	Drug	Cell type	Ref.
Galactose	Asialoglycoprotein receptor (ASGPR)	Lactosylated Liposomal	Dox	HepG2	105
Lactobionic acid		Lactobionic acid liposomes	Oxaliplatin	BEL7402 HCC cell lines	116
Hyaluronic acid	CD44 receptor	HA-functionalized Liposomal	Icaritin	Huh7 and HepG2	106
Folate	Folate receptor	Folic acid-modified PEGylated liposome system	Diacid metabolite of norcantharidin	H22 hepatoma cells	112
Transferrin	Transferrin receptor	Transferrin-modified liposomes	Acetylcholinesterase (AChE)	SMMC-7721	54
Glycyrrhetinic acid	Protein kinase C-α	Glycyrrhetinic acid modified liposomes	Murrayafoline A (MuA)	HepG2	113 and 117

#### 5.2.2. Cell membrane-coated NPs

CM NP formulations have been developed and widely studied in cancer treatment as they can target cancer cells while minimizing recognition by immune cells. Zhang *et al.*'s innovative research examined human hair-derived NPs (HNPs) coated with red blood cell membranes (RBCM) loaded with DSPE-PEG-cRGD peptide for enhanced tumor targeting in liver cancer therapy. DSPE-PEG-cRGD powder was added to isolated RBCM and coated onto HNP using ultrasound, forming membrane-coated HNP@RBCM-cRGD with a mean diameter of 93.51 nm. The biodegradable RBC coating extended the circulation time of the NPs to 24 hours by avoiding macrophage recognition, and minimized off-target effects, enhancing drug efficacy at tumor sites in Hepa 1–6 tumor-bearingmice. Upon irradiation with an 808 nm laser at 1.0 W cm^−2^ for 10 minutes, HNP@RBCM-cRGD raised the tumor temperature to 59.8 °C, outperforming non-coated HNPs, which reached only 51.57 °C, and controls (PBS and RBCM-cRGD), which showed minimal temperature increases below 45 °C, effectively ablating liver cancer cells while sparing healthy tissue.^114^

**Fig. 5:**
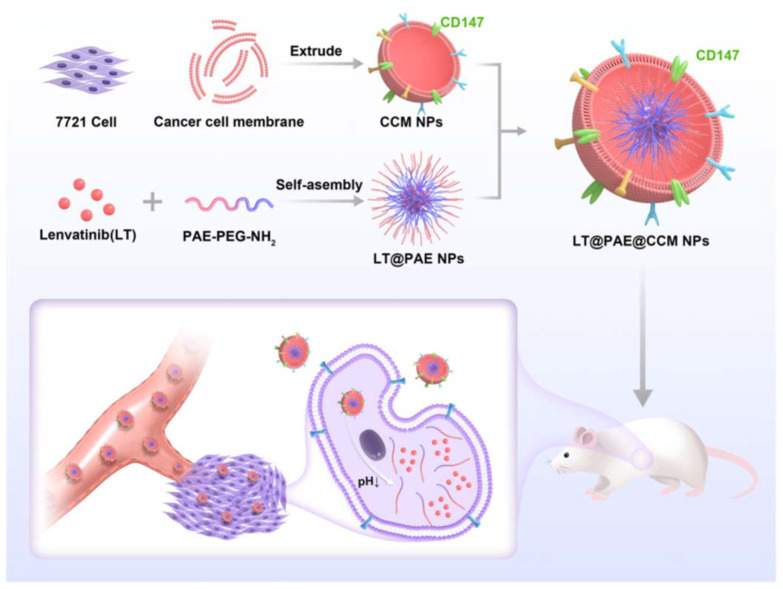
Schematic illustration of cancer cell membrane-camouflaged lenvatinib-encapsulated pH-sensitive polymeric NPs for HCC. The cancer cell membrane provides active homologous targeting to HCC and releases the drug in response to acidic pH at the tumor microenvironment. Reprinted from Wu *et al*.,^115^ under a Creative Commons Attribution (CC BY) license (https://creativecommons.org/licenses/by/4.0/).

In another study by Wu and his group, Lenvatinib was encapsulated within a pH-sensitive polymer and further camouflaged with a cancer cell membrane (Fig. 5). The membrane coating imparted homologous targeting and immune evasion properties to the synthesized NPs, which was confirmed through western blotting and *in vivo* biodistribution studies. There was successful retention of adhesion molecules like EpCAM, galectin-3, and CD-147 on the final NPs, which can be used for homologous binding to the source cells. The live/dead staining showed around 55.3% of death in Lenvatinib-loaded NPs compared to other groups. The anti-tumor properties of lenvatinib-loaded NPs were confirmed *in vivo* showing a significant reduction in tumor weights, which was five times less than other groups.^115^ More recently, Xie *et al.* reported the development of a liver cancer cell membrane-coated iron-based metal organic framework drug delivery system for homologous targeting of cancer cell membranes to overcome hypoxia in the tumor microenvironment. The formulation contained the HIF-1α inhibitor Acriflavine while the metal organic framework was used to catalyze H_2_O_2_ – induced production of OH radicals in the acidic tumor environment. The formulation (∼450 nm diameter) was biocompatible and successfully increased ROS production and inhibited HIF-1α expression *in vitro* in Hepa 1–6 HCC cells and *in vivo* in Hepa 1–6 tumor-bearing nude mice, alleviating tumor hypoxia and demonstrating the potential of this formulation for anti-cancer therapy. In the near future, we can expect to see more formulations incorporating biocompatible lipids and cell membrane-based coatings to address critical challenges in liver cancer therapy.

## 6. Conclusions

Biomimetic NP platforms are an innovative class of drug carriers integrating the biological characteristics of cells and tissues with the improved half-life and sustained/controlled release properties of nanomedicine to deliver therapies in a targeted and effective manner. This review summarizes recent advancements in the application of biomimetic formulations such as liposomes and membrane-coated NPs, specifically in the treatment of liver fibrosis and cancer. These biomimetic nanoplatforms offer great improvement in targeting specificity, immune escape ability, and delivery of multiple therapeutic cargoes for liver disease treatment. The liposomes and CM NPs can accommodate a variety of cargoes, including antifibrotic drugs, genes, ultrasound- or photothermal-responsive agents, and chemotherapeutic drugs. Although liposomes are widely used in drug delivery, their flexibility in terms of surface functionalization is limited when compared to CM NPs, which have membrane proteins that inherently provide cell targeting features. The stealth property of the CM NPs also reduces opsonization, providing prolonged circulation. As biomimetic NPs continue to be increasingly explored in preclinical as well as clinical research, there are several considerations, particularly due to the complexity of the materials used, to be addressed for successful clinical translation. The preparation of liposomes is well established and easier to scale up; there are commercial liposomal formulations already available in the market. However, the process of extracting and coating cell membranes on NPs is more complex. Obtaining sufficient yield of high-quality, functional CMs in an affordable manner is a major barrier to industry-scale production of these formulations. Scale-up of both types of NP formulations while maintaining consistency in size, shape, and functionality is also an important area of focus. Due to the natural membrane coating, membrane-coated NPs can be used in cancer therapy, targeted drug delivery, and immunotherapy. Membranes can be engineered to contain specific biomarkers or membrane proteins for potentially more effective disease detection and treatment than liposomes. Due to their abundance, ease of isolation as well as ability to evade macrophages, RBCMs are widely studied currently as a cell membrane coating material, and they have significant potential for clinical translation.^118^ For large-scale culture of other cell types for cell membrane isolation, bioreactors will need to be used. Following isolation of the cell membranes and coating onto NPs, batch-to-batch consistency in the amount of proteins and lipids present per batch of NPs must be ensured. The NP stability and coating retention under various physiological conditions must also be assessed carefully to ensure that the coating does not degrade before time, affecting their effectiveness in long-term applications. Stability issues and rapid drug release are major barriers to the implementation of liposomes for long-term drug delivery. Previous studies indicate that liposomal stability can decrease when exposed to high temperatures, changes in pH, and physical stress, which can affect drug delivery.^119^ The properties and compatibility of the payload must also be considered while developing biomimetic formulations. It is challenging to encapsulate hydrophobic or large molecules in liposomes as it is difficult to control the drug release rate and may result in premature leakage. Encapsulation of such payloads can be done within polymeric formulations followed by coating with lipids or cell membranes to ensure controlled release of the therapeutic. Liposomal uptake or penetration through some biological barriers is limited and therefore require specialized modifications and formulation optimization for effectiveness.

Detailed preclinical and clinical studies are key to optimize the formulation for the desired applications. Since cell membranes obtained from natural cells are complex due to the presence of multiple proteins and receptors on their surfaces, some of which may not be relevant for the desired application; it is necessary to ensure that these formulations are biocompatible and do not elicit an immune response or cause unintended effects upon administration.^120^ Their long-term safety and biodistribution profile also needs to be carefully assessed in preclinical models and during human clinical trials. Using cell membranes isolated from the patient's own cells for treatment can allay some of these concerns while also offering a pathway for personalized, patient-specific treatment. To further improve NP targeting and treatment efficacy, cell-specific ligands may be embedded into the cell membrane or lipid layer of the biomimetic NPs. The research area of biomimetic NP-based drug delivery systems has received considerable attention in recent years due to their ability to combine stealth with targeted and sustained drug delivery. This review summarizes the applicability of these cutting-edge systems in the treatment of chronic liver disorders such as liver fibrosis and liver cancer, for which there are no effective cures available currently. We can certainly anticipate many more significant breakthroughs using biomimetic formulations in the coming years that will potentially shape the future of liver disease treatment.

## Author contributions

Veena Vijayan: writing – review & editing, writing – original draft, methodology. Janitha Unagolla: writing – original draft. Dhruvisha Panchal: writing – original draft. Judith John: writing – original draft. Siddharth S Menon: writing – original draft. Jyothi U. Menon: writing – review & editing, writing – original draft, funding acquisition.

## Conflicts of interest

The authors declare that they have no known competing financial interests or personal relationships that could have appeared to influence the work reported in this paper.

## Data Availability

No primary research results, software or code have been included and no new data were generated or analysed as part of this review.
